# *Cucumis sativus* Aqueous Fraction Inhibits Angiotensin II-Induced Inflammation and Oxidative Stress In Vitro

**DOI:** 10.3390/nu10030276

**Published:** 2018-02-28

**Authors:** Celeste Trejo-Moreno, Marisol Méndez-Martínez, Alejandro Zamilpa, Enrique Jiménez-Ferrer, Maria Dolores Perez-Garcia, Omar N. Medina-Campos, José Pedraza-Chaverri, María Angélica Santana, Fernando R. Esquivel-Guadarrama, Aida Castillo, Jacquelynne Cervantes-Torres, Gladis Fragoso, Gabriela Rosas-Salgado

**Affiliations:** 1 Instituto de Investigación en Ciencias Básicas y Aplicadas, Universidad Autónoma del Estado de Morelos, Av. Universidad 1001, Cuernavaca, Morelos CP 62209, Mexico; trejomc@hotmail.com (C.T.-M.); mm.mary87@gmail.com (M.M.-M.); 2 Facultad de Medicina, Universidad Autónoma del Estado de Morelos, Leñeros S/N, Cuernavaca, Morelos CP 62350, Mexico; fernando.esquivel@uaem.mx; 3 Laboratorio de Farmacología, Centro de Investigación Biomédica del Sur, Instituto Mexicano del Seguro Social, Republica de Argentina 1, Xochitepec, Morelos CP 62790, Mexico; azamilpa_2000@yahoo.com.mx (A.Z.); enriqueferrer_mx@yahoo.com (E.J.-F.); lola_as@yahoo.com.mx (M.D.P.-G.); 4 Departamento de Biología, Facultad de Química, Universidad Nacional Autónoma de México, Coyoacán, Mexico City CP 04510, Mexico; omarnoelmedina@gmail.com (O.N.M.-C.); pedrazachaverri@gmail.com (J.P.-C.); 5 Centro de Investigación en Dinámica Celular (IICBA), Universidad Autónoma del Estado de Morelos, Av. Universidad 1001, Cuernavaca, Morelos CP 62209, Mexico; santana@uaem.mx; 6 Departamento de Fisiología Biofísica y Neurociencias del Centro de Investigación y Estudios Avanzados del Instituto Politécnico Nacional (CINVESTAV del IPN), Mexico City CP 07360, Mexico; aidacast10@hotmail.com; 7 Departamento de Inmunología, Instituto de Investigaciones Biomédicas, Universidad Nacional Autónoma de México, Coyoacán, Mexico City CP 04510, Mexico; jcervantes@iibiomedicas.unam.mx (J.C.-T.); gladis@unam.mx (G.F.)

**Keywords:** inflammation, ROS, angiotensin II, *Cucumis sativus*

## Abstract

Inflammation and oxidative stress play major roles in endothelial dysfunction, and are key factors in the progression of cardiovascular diseases. The aim of this study was to evaluate in vitro the effect of three subfractions (SFs) from the *Cucumis sativus* aqueous fraction to reduce inflammatory factors and oxidative stress induced by angiotensin II (Ang II) in human microvascular endothelial cells-1 (HMEC-1) cells. The cells were cultured with different concentrations of Ang II and 0.08 or 10 μg/mL of SF1, SF2, or SF3, or 10 μmol of losartan as a control. IL-6 (Interleukin 6) concentration was quantified. To identify the most effective SF combinations, HMEC-1 cells were cultured as described above in the presence of four combinations of SF1 and SF3. Then, the effects of the most effective combination on the expression of adhesion molecules, the production of reactive oxygen species (ROS), and the bioavailability of nitric oxide (NO) were evaluated. Finally, a mass spectrometry analysis was performed. Both SF1 and SF3 subfractions decreased the induction of IL-6 by Ang II, and C4 (SF1 and SF3, 10 μg/mL each) was the most effective combination to inhibit the production of IL-6. Additionally, C4 prevented the expression of adhesion molecules, reduced the production of ROS, and increased the bioavailability of NO. Glycine, arginine, asparagine, lysine, and aspartic acid were the main components of both subfractions. These results demonstrate that C4 has anti-inflammatory and antioxidant effects.

## 1. Introduction

Inflammation and oxidative stress are two significant hallmarks of endothelial dysfunction and play a critical role in the pathogenesis of circulatory disorders such as hypertension, coronary artery disease, chronic heart failure, peripheral artery disease, and chronic renal failure. These diseases are the main causes of morbidity and mortality in the world [1]. Angiotensin II (Ang II) has been implicated in the pathology of endothelial dysfunction as a source of inflammation and oxidative stress [2,3]. Ang II binds to type-1 angiotensin receptors (AT1R) on endothelial cells and promotes the activation of nicotinamide adenine dinucleotide (NADH) oxidase, which in turn increases the levels of superoxide anion (O_2_^∙−^), a reactive oxygen species (ROS) that decreases the bioavailability of nitric oxide (NO), which is necessary to maintain vascular tone [4,5]. Additionally, O_2_^∙−^ uncouples endothelial nitric oxide synthase (eNOS) and oxidizes tetrahydrobiopterin (BH4), its cofactor, leading to further production of O_2_^∙−^ instead of NO [6].

The presence of ROS is detected by proteins sensitive to the redox status of the cell; in turn, these proteins activate AKT (Protein kinase B), MAPKs (mitogen-activated protein kinases), and NF-κB (Nuclear factor kappa B) [7,8,9,10], triggering inflammation and promoting the synthesis of IL-1β, IL-6, TNF-α, C reactive protein, E-selectin, ICAM-1 (Intercellular Adhesion Molecule 1), VCAM-1 (Vascular cell adhesion protein 1), and MCP-1 (Monocyte chemoattractant protein-1), among other molecules [11,12,13,14].

Plant-derived products are attractive sources of new active ingredients for medicines, pharmacological tools, and to control various diseases improving the quality of life of patients suffering from long-lasting diseases [15]. Cucumber (*Cucumis sativus*) is a member of the Cucurbitaceae family, which includes species with therapeutic potential such as melon, squash, and pumpkin. It is a popular crop used in Indian traditional medicine since ancient times. Traditionally, this plant has been used to treat headaches and hyperlipidemia, and to prevent constipation. Seeds and the fruit have refreshing properties, soothing irritated skin and reducing swelling [16,17]. Moreover, cucumber has been reported to have antiinflammatory and antioxidant properties [17,18]. This study was aimed to evaluate the in vitro effect of the SF1, SF2, and SF3 subfractions from the *C. sativus* aqueous fraction to downregulate the inflammatory and oxidative effects induced by Ang II in human endothelial cells, and to identify the molecules potentially involved in these effects. SF1 and SF3, either alone or in combination, significantly downregulated the inflammatory and oxidative effects induced by Ang II in endothelial cells. Amino acids such as glycine, arginine, asparagine, lysine, and aspartic acid seem to be responsible for this effect.

## 2. Material and Methods

### 2.1. Plant Material and Cucumis sativus Subfractions

*Cucumis sativus* aerial parts, including fruits, were collected from an edible crop free from pesticides and fertilizers in Xochitepec, Mexico, from July through August. The plant material was kept away from light at room temperature and dried in an oven at 50 °C for 36 h. Extracts were obtained immediately once the material was completely dried. Dry plant material was ground in a Pulvex electric mill (Büchi R-114, Büchi Labortechnik, Flawil, Switzerland) until particles smaller than 4 mm were obtained. An exhaustive maceration process with ethanol/water (60:40 *v*/*v*) was performed at room temperature to obtain the hydroalcoholic extract. This extract was concentrated by reduced-pressure distillation under controlled temperature and then lyophilized. A sample of this extract (50 g) underwent bipartition with ethyl acetate/water, obtaining the aqueous fraction. This fraction was concentrated by reduced-pressure distillation under controlled temperature. Thirty-five grams of the *Cucumis sativus* aerial parts, including fruits, were collected from an edible crop free from pesticides and fertilizers in Xochitepec from July through August. The plant material was kept away from light at room temperature and dried in an oven at 50 °C for 36 h; aqueous fractions were suspended in methanol (700 mL) for 24 h; the liquid phase was filtered and concentrated in a rotary-evaporator (Laborota 4000, Heidolph, Schwabach, Germany), yielding the subfraction SF1, which was dried by lyophilization. On the other hand, the organic phase was suspended in acetone (700 mL), yielding the subfractions SF2 (soluble phase) and SF3 (precipitate), which finally were concentrated under reduced pressure and lyophilized.

### 2.2. Thin-Layer Chromatography

To estimate the chemical profile of the subfractions under study, normal- and reverse-phase thin-layer chromatography (TLC) assays were performed using commercially available standards (rutin, quercetin, glucose, glycine, alanine, serine, valine, leucine, asparagine, aspartic acid, lysine, glutamic acid, and arginine). The systems dichloromethane:methanol (7:3 *v*/*v*), ethyl acetate:methanol:water:glacial acetic acid (7:2:2:0.5 *v*/*v*/*v*/*v*), and *n*-butanol:acetone:glacial acetic acid:water (35:35:10:20 and 70:70:20:10 *v*/*v*/*v*/*v*) were used as solvents for normal-phase TLC, while water:acetonitrile (7:3 *v*/*v*) was used for reverse-phase TLC. Once the plates were developed, spots were visualized with 4-hydroxybenzaldehyde (Merck, Darmstadt, Germany) and aminoethanol dimethylborate (Sigma-Aldrich, St. Louis, MO, USA) for flavonoids, and ninhydrin (Merck) for amino acids, following the supplier’s directions.

### 2.3. Mass Spectrometry

To identify the compounds responsible for the biological activities of SF1 and SF3, a mass spectrometry analysis was performed at the facilities of the Centro de Investigaciones Biomédicas del Sur (CIBIS, Xochitepec, Mexico). A 500 µg sample was diluted in trifluoracetic acid 0.05% to a final concentration of 50 µg/mL. Glycine, arginine, lysine, leucine, isoleucine, aspartate, and glutamate (Sigma) diluted to the same concentration were used as standards. All samples were analyzed in a triple quadrupole TQD mass spectrometer (Waters, Milford, MA, USA) coupled to an Acquity liquid chromatograph (Waters) through a combined electrospray-APCI Z-spray ion source. All samples were analyzed in positive and negative ion modes. Finally, amino acids were identified in the samples by mass spectra comparison with the standards.

### 2.4. Cell Culture

Human microvascular endothelial cells-1 (HMEC-1) were provided by Aida Castillo from the Department of Physiology, Biophysics and Neurosciences, CINVESTAV-IPN, México City. The cells were cultured in MCDB-131 medium supplemented with fetal serum bovine (FBS) %, l-glutamine 10 mM, 100 U/mL of penicillin-streptomycin (Invitrogen, Carlsbad, CA, USA), 10 ng/mL of endothelial growth factor, and 1 μg/mL of hydrocortisone (Sigma) at 37 °C under a CO_2_ atmosphere (5%). All experiments were performed using passages 3 to 8. The cells were cultured in 24-well plates to a density of 5 × 10^5^ cells/well in 500 μL of medium supplemented with Ang II at a concentration of 0, 8, 40, 200, 1000, or 5000 nM and 0.08 or 10 μg/mL of SF1, SF2, or SF3, either for 12 h to evaluate the antiinflammatory effect, or for 6 h to evaluate the antioxidant profile of the subfractions. As a control, HMEC-1 cells were treated with 10 μmol of losartan [19] instead of the subfractions. In some experiments, the subfractions SF1 and SF3 were combined at a concentration of 0.08 μg/mL each (C1), SF1 0.08 μg/mL and SF3 10 μg/mL (C2), SF1 10 μg/mL and SF3 0.08 μg/mL (C3), and SF1 and SF3 (10 μg/mL each) (C4). For ROS and NO determination, the cells were stimulated with Ang II 5000 nM.

### 2.5. Immunocytochemistry

HMEC-1 cells were cultured on poly-l-lysine-coated coverslips in 24-well plates under the conditions described above. By the end of culture time, the cells were washed three times with PBS (Phosphate Buffer solution) (NaCl 140 mM, KCl 2 mM, and K_2_HPO_4_ 1.15 mM) and fixed in acetone for 15 min. Endogenous peroxidase was blocked with H_2_O_2_ 30% for 10 min. Then, the cells were incubated with PBS-BSA (Bovine Serum Albumin) 0.1%-Tween 20 0.05% (Sigma) to block unspecific binding sites. The cells were incubated with primary mouse anti-human anti-ICAM-1 and anti-E-selectin antibodies (1:100 and 1:50, respectively) (eBioscience, Waltham, MA, USA) overnight at 4 °C. The cells were washed with PBS and incubated with goat anti-mouse biotinylated IgG antibody (1:500) (Santa Cruz Biotechnology, Dallas, TX, USA) for 30 min at 37 °C, followed by incubation with HRP (horseradish peroxidase)-streptavidin (eBioscience). The chromogenic substrate 3,3′-diaminobenzidine (BioCare Medical, Pacheco, CA, USA) was added, and 5 min later the reaction was stopped by washing the slides with water. Then, the cells were counterstained with hematoxylin. Images were obtained with an ECLIPSE 80i microscope (Nikon, Tokyo, Japan).

### 2.6. Western Blot

HMEC-1 cells were washed with PBS and lysed by incubating with ice-cold RIPA (Radioimmunoprecipitation assay) lysis buffer (Tris 20 mM, pH 7.4; NaCl 150 mM, EDTA (Ethylenediaminetetraacetic acid) 1 mM, pH 7.4; Triton X-100 0.5%; SDS (Sodium dodecyl sulfate) 0.1%, sodium deoxycholate 0.5%), using a cocktail of phosphatase and protease inhibitors (sodium fluoride (NaF) 25 mM; sodium pyrophosphate (NaPPi) 1 mM; sodium vanadate (NaVPO_4_) 1 mM; phenylmethylsulfonyl fluoride (PMSF) 1 mM; pepstatin A 0.1 mg/mL; leupeptin 0.1 mg/mL; antipain 0.1 mg/mL; aprotinin 0.1 mg/mL) (Sigma) for 15 min. The lysates were scraped off the plates and centrifuged at 13,000 rpm for 10 min at 4 °C. Proteins in the supernatant were quantified by the Lowry method. Twenty micrograms of protein were denatured by boiling for 5 min in sample buffer, separated by SDS-PAGE (polyacrylamide gel electrophoresis) in acrylamide 10% gels and electro-transferred to a polyvinylidene difluoride (PVDF) membrane (Merck) using transfer buffer (Tris 25 mM, pH 8.5; glycine 193 mM; methanol 20%). Membranes were blocked for 1 h in blocking buffer TBS-T (Tris-buffered saline containing Tween-20) 0.1%) with BSA. 2%. Thereafter, the membranes were incubated overnight with primary anti-E-selectin (Invitrogen) or anti-α-actin (Biolegend, San Diego, CA, USA) antibodies diluted 1:1000 or 1:3000, respectively, in blocking buffer. After washing three times for 5 min with TBS-T, the blots were incubated with goat anti-mouse (Thermo Scientific, Waltham, MA, USA) or anti-rabbit (Abcam, Cambridge, UK) antibodies, diluted 1:2500. Antibody binding was detected with the SuperSignal West Dura Extended Duration Substrate solution (Thermo Scientific). The bands were analyzed using the ImageJ software (National Institute of Health, Bethesda, MD, USA) and the anti-actin signal was used for data normalization.

### 2.7. ROS Quantification

Intracellular ROS levels were quantified by dihydroethidium (DHE), which is oxidized by superoxide to yield ethidium; the latter binds nuclear DNA and emits a red fluorescence (535 nm Ex; 610 nm Em). The substrates NADH (1 mM), succinate (5 mM), l-arginine (1 mM), and xanthine (1 mM) were added to identify ROS sources, along with the inhibitors: DPI (Diphenyleneiodonium) (0.1 mM) to inhibit NADPH (nicotinamide adenine dinucleotide phosphate) oxidase; antimycin (0.05 mM) for mitochondrial complex II; l-NAME (N(ω)-nitro-l-arginine methyl ester) (1 mM) for eNOS; and allopurinol (0.02 mM) for xanthine oxidase.

Fifteen minutes later, the cells were incubated with DHE 20 µM and washed three times with warm PBS. Then, the cells were placed in fresh medium, analyzed, and photographed in a Cytation 5 cell imagen multimodal plate reader (Biotek Instruments, Winooski, VT, USA), using the Gen 5 software (Biotek Instruments) under a 20× objective. Finally, the intensity of DHE emission was quantified with Metamorph v. 6.1 (Molecular Devices, San Jose, CA, USA).

### 2.8. ELISA

Cell culture medium was collected after a 12-h culture, and IL-6 concentration was measured by ELISA (OptEIA kit BD-Biosciences, Franklin Lakes, NJ, USA) following the manufacturer’s directions. Briefly, 96-well plates were sensitized with the capture antibody overnight at 4 °C. The plates were blocked with PBS-FBS 10% for 1 h at room temperature. Then, either medium or the IL-6 standard was added and incubated at room temperature for 2 h, followed by incubation for 1 h at room temperature with the horseradish peroxidase-coupled detection antibody. The chromogenic substrate tetramethylbenzidine (TMB) (Invitrogen) was added, and the reaction was stopped 30 min later with H_2_SO_4_ 2N. Absorbance was determined at 450 nm at 37 °C using a VERSAmax microplate reader (Molecular Devices, San Jose, CA, USA). IL-6 concentration was calculated according to a standard curve.

The results obtained were used to plot a concentration–response curve and to determine the maximal effect (*E*_max_) and effective 50% concentration (EC_50_) values for each subfraction.

### 2.9. NO Quantification

NO levels were measured as nitrite/nitrate (final products of NO metabolism) by the Griess reaction [20]. A 100-μL aliquot of the medium was placed in 96-well plates and incubated with 100 μL of Griess reagent ((1-naphthyl)-ethylenediamine 0.1% and 1% sulfanilamide in phosphoric acid 2.5%) for 30 min at room temperature. Absorbance was measured at 540 nm in a VERSAmax microplate reader (Molecular Devices). The amount of NO in each sample was determined by a sodium nitrite standard curve.

### 2.10. Statistical Analysis

Data are reported as mean ± SD (standard deviation). Significant differences among conditions were determined by ANOVA and the post-hoc Tukey test, with a significance value of *p* < 0.05.

## 3. Results

### 3.1. SF1 and SF3 Controlled IL-6 Production

**Figure 1:**
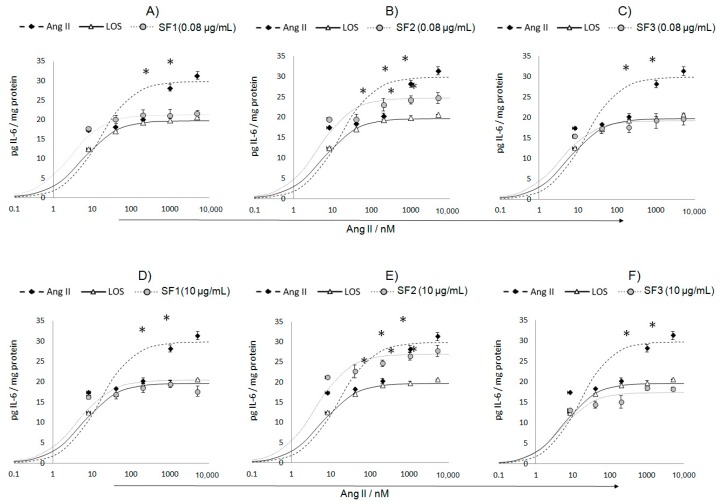
Concentration–response curves of the subfractions evaluated on Ang II (angiotensin II)-induced IL-6 (Interleukin 6) production, 12 h after treatment. (**A**–**C**) Effect of 0.08 μg/mL of SF1 (subfraction 1), SF2, and SF3; (**D**–**F**) Effect of 10 μg/mL of SF1, SF2, and SF3. Human microvascular endothelial cells-1 (HMEC-1) cells were co-cultured with the concentrations of Ang II specified above. Losartan (LOS) was used as a positive control. Data are reported as mean ± SD (standard deviation) and were analyzed by ANOVA and the post-hoc Tukey test. *n* = 5, * *p* < 0.05 vs. control (Ang II-free).

As seen in Figure 1, the production and release of IL-6 by endothelial cells exposed to the Ang II stimulus showed a concentration-dependent behavior, *E*_max_ = 31.4 pg/mg and EC_50_ = 14.9 nM. Treatment with losartan, a pharmacological antagonist (a competitive inhibitor of AT1R), decreased the values of the pharmacological constants of Ang II, *E*_max_ = 19.7 pg/mg and EC_50_ = 5.8 nM. On the other hand, both concentrations (0.08 and 10 μg/mL) of SF1 and SF3 antagonized the effect of Ang II: *E*_max_ = 21.3 pg/mg and EC_50_ = 5.8 nM (panel A); *E*_max_ = 20.4 pg/mg and EC_50_ = 4.3 nM (panel D); *E*_max_ = 19.2 pg/mg and EC_50_ = 3.5 nM (panel C); and *E*_max_ = 17.4 pg/mg and EC_50_ = 4.9 nM (panel F). Finally, SF2 failed to control IL-6 production in any of the assessed concentrations: *E*_max_ = 24.7 pg/mg and EC_50_ = 4.4 nM (panel B), and *E*_max_ = 27.0 pg/mg and EC_50_ = 4.0 nM (panel E).

### 3.2. C4 Is the Best Combination to Control IL-6 Production

**Figure 2:**
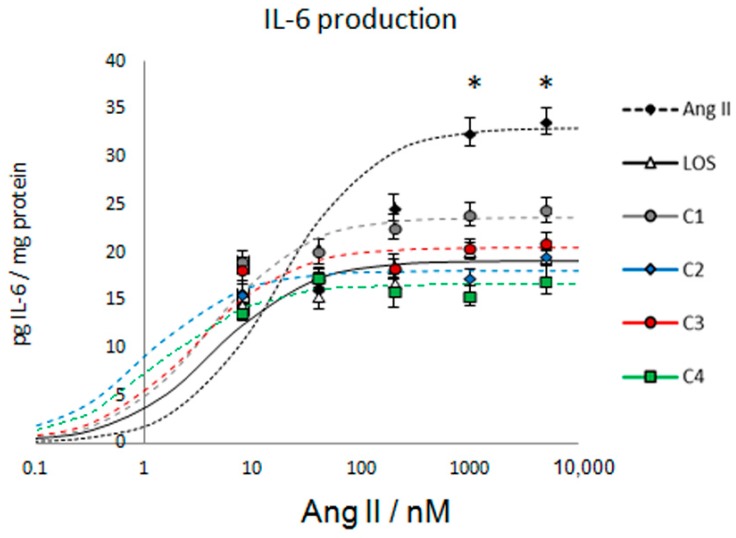
Concentration–response curves of the assessed combinations on Ang II-induced IL-6 production, 12 h after treatment. Losartan (LOS) was used as a positive control. C1 = 0.08 μg/mL of SF1 and 0.08 μg/mL of SF3; C2 = 0.08 μg/mL of SF1 and 10 μg/mL of SF3; C3 = 10 μg/mL of SF1 and 0.08 μg/mL of SF3; C4 = 10 μg/mL of SF1 and 10 μg/mL of SF3. Data are reported as mean ± SD and were analyzed by ANOVA and the post-hoc Tukey test. *n* = 5, * *p* < 0.05 vs. control (Ang II-free).

To identify the most effective combination of the subfractions to control the proinflammatory status, HMEC-1 cells were cultured with Ang II and four different combinations of SF1 and SF3. Concentration–response curves showed an *E*_max_ = 33.0 pg/mg and EC_50_ = 16.9 nM for Ang II; losartan antagonized the effect of Ang II, *E*_max_ = 19.2 pg/mg and EC_50_ = 4.4 nM (Figure 2). All four combinations decreased the value of these pharmacological constants; nevertheless, C4 was the most effective: *E*_max_ = 23.6 pg/mg and EC_50_ = 4.3 nM (panel A); *E*_max_ = 18.1 pg/mg and EC_50_ = 3.1 nM (panel B); *E*_max_ = 20.5 pg/mg and EC_50_ = 3.9 nM (panel C); and *E*_max_ = 16.7 pg/mg and EC_50_ = 2.9 nM (panel D).

### 3.3. C4 Prevented E-Selectin and ICAM-1 Expression

**Figure 3:**
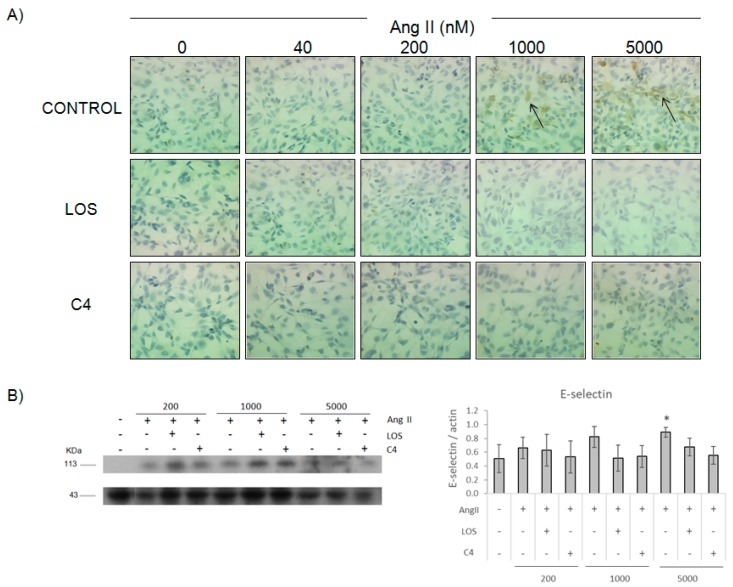
Effect of C4 on Ang II-induced E-selectin expression, 12 h after treatment. Immunocytochemistry (**A**). Western blot and expression relative to actin (**B**). Arrows indicate the E-selectin label. Microphotographs were taken with a 40× objective. LOS: Losartan; C4: Combination of SF1 and SF3, 10 μg/mL each. Data are reported as mean ± SD and were analyzed by ANOVA and the post-hoc Tukey test. *n* = 4, * *p* < 0.05 vs. control (Ang II-free).

Along with cytokine production, a well-known proinflammatory parameter induced by Ang II is the expression of adhesion molecules. Thus, the capacity of C4 to prevent the expression of E-selectin (Figure 3) and ICAM-1 (Supplementary Figure S1) was evaluated. Our results indicate that HMEC-1 cells expressed both adhesion molecules in the presence of Ang II 1000 and 5000 nM. This expression was diminished by C4, even at the highest Ang II concentration tested. Similar results were obtained with losartan.

### 3.4. C4 Favored NO Bioavailability

**Figure 4:**
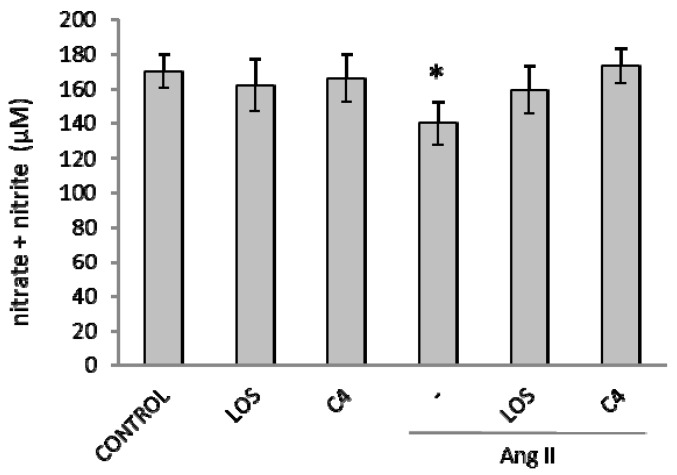
Effect of C4 on nitric oxide (NO) availability. HMEC-1 cells were stimulated with LOS or C4 or with Ang II 5000 nM plus LOS or C4. Data are reported as mean ± SD (standard deviation) and were analyzed by ANOVA and the post-hoc Tukey test. *n* = 5, * *p* < 0.05 vs. control (Ang II-free). LOS: Losartan; C4: Combination of SF1 and SF3, 10 μg/mL each.

The decrease in NO bioavailability is a key marker of oxidative stress induced by Ang II. Thus, we assessed the capacity of C4 to prevent a reduction in NO availability by the Griess reaction. As shown in Figure 4, Ang II caused a small but significant reduction in NO bioavailability; this effect was abolished by C4, maintaining this parameter at levels similar to Ang II-free and losartan controls.

### 3.5. C4 Prevented Ang II-Induced ROS Increase

**Figure 5:**
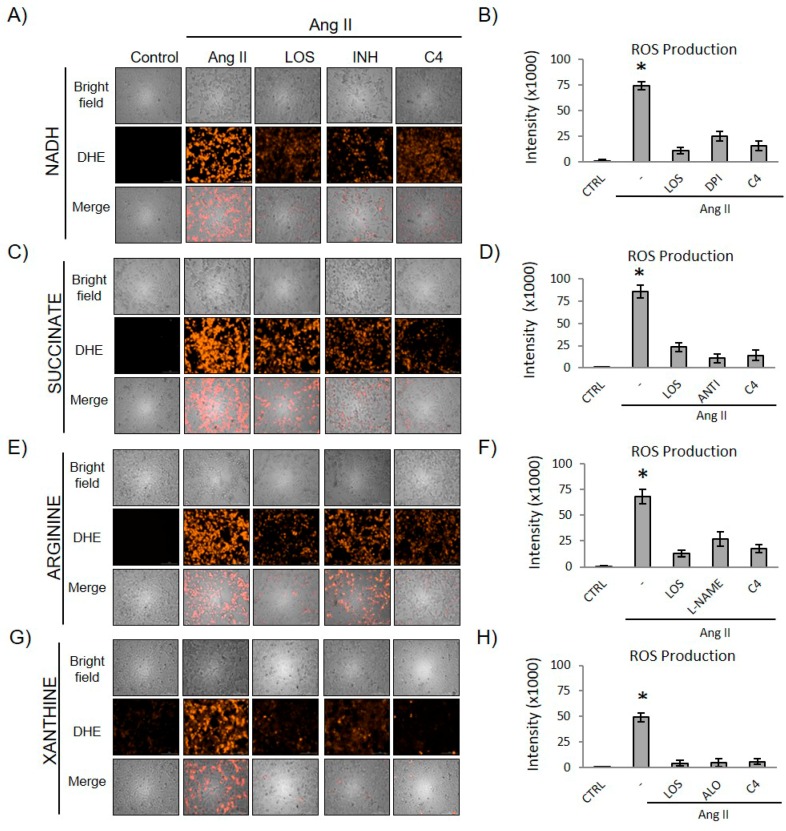
Effect of C4 on the production of O_2_^∙−^ from four different reactive oxygen species (ROS) sources, NADH (**A**,**B**), Succinate (**C**,**D**), Arginine (**E**,**F**) and Xanthine (**G**,**H**). HMEC-1 cells were stimulated with Ang II 5000 nM for 6 h. Representative microphotographs were taken with a 20× objective (**A**,**C**,**E**,**G**). Data are reported as mean ± SD and were analyzed by ANOVA and the post-hoc Tukey test. *n* = 5, * *p* < 0.05 vs. control (Ang II-free), (**B**,**D**,**G**,**H**). LOS: Losartan; INH: Respective inhibitor; C4: Combination of SF1 and SF3, 10 μg/mL each; DHE: Dihydroethidium; ROS: reactive oxygen species; ALO: allopurinol; CTRL: Control; DPI: Diphenyleneiodonium; NADH: nicotinamide adenine dinucleotide phosphate; ANTI: antimycin; l-NAME: N(ω)-nitro-l-arginine methyl ester.

Ang II promotes oxidative stress by inducing the activation of enzymes such as NADH oxidase, the respiratory chain, eNOS, and xanthine oxidase. Thus, we evaluated the effect of C4 on the production of ROS by these enzymes. As shown in Figure 5, Ang II significantly increased the generation of ROS by the four enzymes assessed. C4 significantly prevented ROS formation, reducing the levels of NADH oxidase-derived ROS by 78.3%, a value similar to those obtained with losartan (85.5%) and the selective inhibitor DPI (66.1%; Figure 5, panels A,B). With respect to mitochondrial ROS, C4 decreased their levels by 83.4%, while the decrease due to losartan was 72.6%, and that due to antimycin was 87.1% (Figure 5, panels C,D). Additionally, C4 reduced ROS production by eNOS (74.5%; Figure 5, panels E,F) and xanthine oxidase (87.7%; Figure 5, panels G,H) to a similar extent as losartan (81.3% and 90.9%), l-NAME (60.7%), and allopurinol (89.5%).

### 3.6. Identification of Major Compounds

To identify the compounds in SF1 and SF3 with potential biological effects, a thin-layer chromatography assay was performed. The results (Supplememtary Figure S2) indicated that amino acids are the major compounds in the subfractions. Mass spectrometry results are shown in Table 1 and Supplememtary Figure S3. Positive ionization produced precursor peaks with molecular weights of 58.84, 133.93, 146.97, and 157.01 Da, matching the glycine, aspartic acid, lysine, and arginine standards, respectively, being the former the major amino acid in both subfractions. On the other hand, negative ionization produced a precursor peak with a molecular weight of 132.98 Da, corresponding to the asparagine standard.

## 4. Discussion

Inflammation and oxidative stress are strongly associated with the progression of endothelial dysfunction; in turn, the latter is the physiopathological substrate of several cardiovascular diseases with high impact on mortality indices such as myocardial infarction, cerebrovascular disease, and chronic renal failure [33,34,35]. Thus, treatments to control both conditions are emerging as key therapies against these high-impact diseases [1].

Whilst endothelial dysfunction was induced in HMEC-1 cells by adding Ang II to the culture medium in concentrations much larger than physiological levels, these concentrations proved to be the most effective conditions to induce endothelial dysfunction in vitro in this cell line. Several studies have used this Ang II dose-range to induce inflammatory and oxidative responses [36,37,38,39,40]. On the other hand, HMEC-1 cells were chosen as a model because they are employed in various studies to induce endothelial dysfunction, increasing the production of ROS and inflammatory factors [41,42,43].

The subfractions SF1, SF2, and SF3 from the *C. sativus* aqueous fraction were evaluated herein for their capacity to prevent Ang II-induced inflammation and oxidative stress in vitro. Losartan, an AT1R antagonist, is capable of inhibiting the downstream effects of its activation. Our findings indicate that SF1 and SF3 modulated several inflammatory and oxidative parameters as effectively as losartan. These results could be due to glycine and arginine, the main compounds detected in SF1 and SF3. Both amino acids have been reported to have anti-inflammatory effects since they inhibit the expression of IL-6, IL-1β, IL-17, TNF-α, and cyclooxygenase 2, as well as macrophage infiltration; they also downregulate the activity of NF-κB [21,22,23,24,29,30,31]. However, as shown by chromatography, other compounds, related to polysaccharides, were also present in the three subtractions; these compounds could also be exerting anti-inflammatory and anti-oxidant effects as observed in other plants of the family Cucurbitaceae [44,45]. Thus, the overall effect of the assayed subfractions on endothelial dysfunction could be due to the combined effect of amino acids and polysaccharides present in these subfractions.

Ang II is an octapeptide; upon binding the receptor AT1R in the endothelial cell, it promotes the MAPK signaling cascade that leads to the activation of the inhibitor of NF-κB (IκB) kinase (IKK); in turn, IKK phosphorylates IκB. After being phosphorylated, IκB is degraded and NF-κB translocates into the nucleus. NF-κB is a transcription factor that controls the expression of genes involved in inflammation such as IL-6, E-selectin, and ICAM-1 [46]. It has been reported that when glycine binds its receptor (GlyR), which is an ionotropic or ligand-gated receptor, it contributes to hyperpolarizing cells such as macrophages, hindering its activation [25]. Other authors [21,47] have reported that this hyperpolarization inhibits the MAPK pathway. Then, the activation of NF-κB induced by Ang II and its inflammatory consequences are blocked. GlyR is also expressed in endothelial cells [48,49]. Thus, glycine in SF1 and SF3 could interact with GlyR in endothelial cells, hyperpolarizing their membrane and blocking the Ang II-mediated MAPK signaling, and consequently inhibiting the activation of NF-κB and the production of IL-6, E-selectin, and ICAM-1. l-Arginine is another amino acid which has exhibited anti-inflammatory properties in vivo and in vitro by diminishing the expression of inflammatory cytokines [29,50]. Recently, l-arginine was demonstrated to inhibit NF-κB activation in Caco-2 cells by a mechanism involving its active transport by the cationic amino acid transporter CAT1. l-arginine also promotes the activity of eNOS to generate NO, increasing its bioavailability [50].

Ang II also has prooxidant effects and induces a decrease in NO bioavailability [5,13]. Upon binding AT1R, it triggers the signaling pathway that activates NADH oxidase to produce O_2_^∙−^ [4], increasing the intracellular concentration of this radical ion, and overcoming cellular antioxidant mechanisms (superoxide dismutase, catalase, and glutathione peroxidase). The increase of ROS also results in eNOS uncoupling, which switches its activity on the same substrate to produce further O_2_^∙−^ instead of NO, increasing the prooxidative environment [6]. The results herein reported demonstrated that a combination of SF1 and SF3 (C4), 10 μg/mL each prevented the decrease in NO levels. Glycine and arginine, the main amino acid components in both subfractions, could be responsible for this effect along with the polysaccharide content (which could not be identified). Glycine has been reported to increase the expression of eNOS mRNA and of the protein itself; thus, more enzyme is available to produce NO [23,26]. On the other hand, the substrate of eNOS is arginine [29]; consequently, SF1 and SF3 could act by simultaneously increasing the levels of the enzyme and its substrate to synthesize NO. In addition, arginine stimulates the phosphoinositide 3-kinase/Akt pathway [30], which favors eNOS activation, the production of NO, and its vasodilatory effects, thus improving endothelial function. Besides, exogenously administered l-arginine has proved to be able to attenuate Ang II-mediated hypertension in rats, reversing the vasoconstrictor effects of Ang II and restoring renal excretory function, probably by mechanisms involving a cellular increase of l-arginine uptake and NO production [51]. Although both amino acids have been reported to exert anti-inflammatory and anti-oxidant effects, the particular combination of each one, along with the presence of other (not yet identified) trace components must be relevant to the overall effect herein reported, since not all combinations were equally efficient to control endothelial dysfunction. Indeed, the C4 combination was the most effective in controlling IL-6 production, a cytokine well known for its capacity to promote inflammation. Human supplementation with any of these two amino acids has been demonstrated to modulate vascular dysfunction [52,53], and these results may support the potential use of a standardized *C. sativus* fraction to control these pathological conditions.

As previously mentioned, Ang II promotes oxidative stress by increasing ROS production. There are many potential sources of ROS in cells, including NADH oxidases, mitochondria, xanthine oxidase, and the impaired eNOS [4]. We showed that treating HMEC-1 cells with C4 (a combination of SF1 and SF3) significantly decreased the Ang II-induced ROS production from these sources. These results could be due to the effect of the amino acid constituents of C4 to improve antioxidant defenses. Arginine and glycine have been reported to increase the amount of copper/zinc superoxide dismutase (Cu/Zn-SOD), an antioxidant enzyme capable of reducing the superoxide ion to hydrogen peroxide [26,30]. On the other hand, arginine has a proven antioxidant activity by its capacity to scavenge free radicals such as 2,2-diphenyl-1-picrylhydrazyl (DPPH) [26]. In addition, glycine is a precursor of glutathione (glutamyl-cysteinyl-glycine), a tripeptide with antioxidative effects mediated by (i) a peroxidase-coupled reaction; (ii) regulation of the cellular sulfhydryl and redox balance; and (iii) regulation of the expression/activation of redox-sensitive transcription factors [24,32].

In conclusion, the particular combination of different concentrations of glycine, arginine, and polysaccharides present in the aqueous fractions of *C. sativus* could be potentially useful to treat various pathologies associated with inflammation and oxidative stress, such as endothelial dysfunction.

## 5. Conclusions

Overall, this study demonstrates that the combination C4 from the SF1 and SF3 subfractions of the *C. sativus* aqueous fraction was efficient in reducing Ang II-induced inflammatory factors and oxidative stress in HMEC-1 cells.

## Figures and Tables

**Table 1:** Amino acids identified in the subfractions SF1 and SF3 from *Cucumis sativus* aqueous fraction by mass spectrometry.

Ion Mode	Precursor Peak (*m*/*z*)	Amino Acid	Molecular Formula	Reported Bioactivity	References
Positive	58.84–58.88	Glycine	C_2_H_5_NO_2_	Reduces TNF-α, IL-6, COX-2, and NF-κB. Improves the bioavailability of NO. Increases GSH, SOD, and eNOS.	[21,22,23,24,25,26,27]
Negative	132.98	Asparagine	C_4_H_8_N_2_O_3_	Downregulates caspase-3, TNF-α, and TLR4 and its downstream signaling.	[28]
Positive	133.93–133.96	Aspartic acid	C_4_H_7_NO_4_	No activity reported.	-
Positive	146.97–147.00	Lysine	C_6_H_14_N_2_O_2_	No activity reported.	-
Positive	175.01–175.06	Arginine	C_6_H_14_N_4_O_2_	NO precursor. Reduces IL-17 and NF-κB. Inhibits iNOS. Increases SOD and PI3K. Scavenger activity.	[29,30,31,32]

TNF-α: tumor necrosis factor α; IL-6: Interleukin 6; COX-2: Cyclooxygenase-2; NF-κB: Nuclear factor kappa B; NO: nitric oxide; GSH: Glutathione; SOD: superoxidase dismutase; eNOS: endothelial nitric oxide synthase; TLR4: Toll-like receptor 4; iNOS: Inducible nitric oxide synthase; PI3K: phosphoinositide 3-kinase.

## Supplementary Materials:

The following are available online at http://www.mdpi.com/2072-6643/10/3/276/s1, Supplementary Figure S1. Effect of C4 on Ang II-induced ICAM-1 expression, 12 h after treatment. Supplementary Figure S2. Thin-layer chromatography. Supplementary Figure S3. Mass spectrometry.
