# Supplementary material for: Cucumis sativus Aqueous Fraction Inhibits Angiotensin II-Induced Inflammation and Oxidative Stress In Vitro
Source: Nutrients. 2018 Feb 28;10(3):276. doi: 10.3390/nu10030276 (PMC5872694; doi:10.3390/nu10030276)
Supplement: Supplementary file 1 [file nutrients-10-00276-s001.pdf]

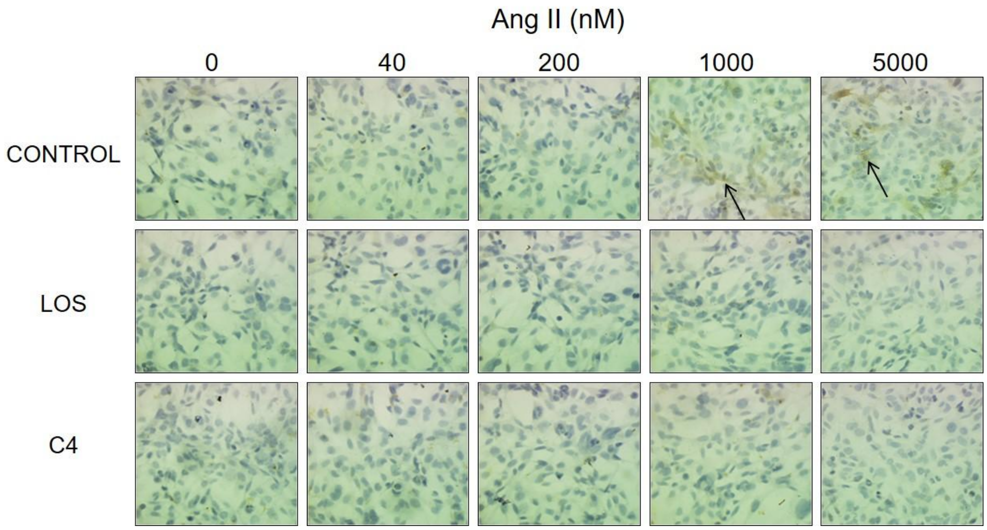

Supplementary Figure 1. Effect of C4 on Ang II-induced ICAM-1 expression, 12 hrs after treatment. Arrows indicate the ICAM-1 label. Microphotographs taken with a 40X objective. LOS = Losartan; C4 = Combination of SF1 and SF3, 10 µg/mL each.

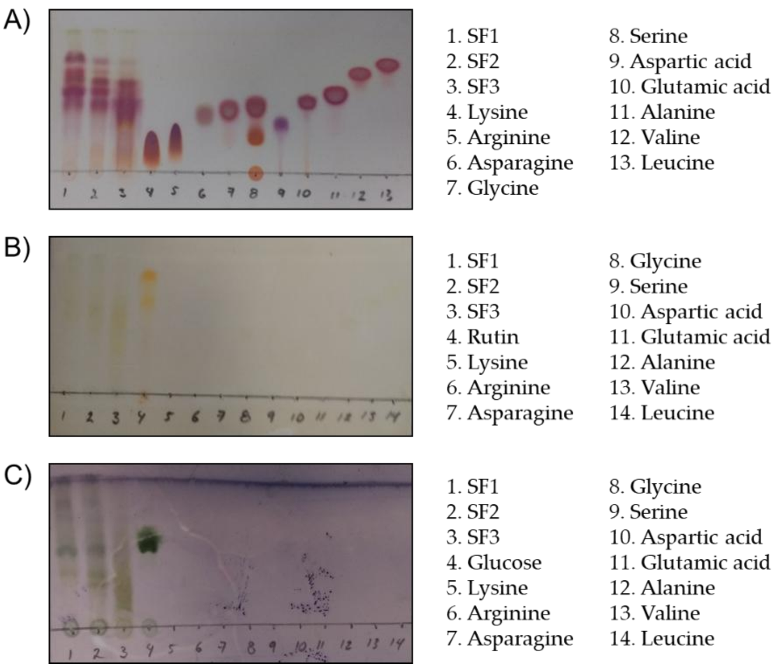

Supplementary Figure 2. Thin-layer chromatography. Separation of compounds in the subfractions of *Cucumis sativus using* normal-phase TLC and the system *n*-butanol: acetone: glacial acetic acid: water (35: 35: 10: 20 v/v). The spots were sprayed with ninhydrin to visualize amino acids (A), aminoethanol dimethylborate to visualize flavonoids (B), and 4-hydroxybenzaldehyde to visualize polysaccharides (C).

A)

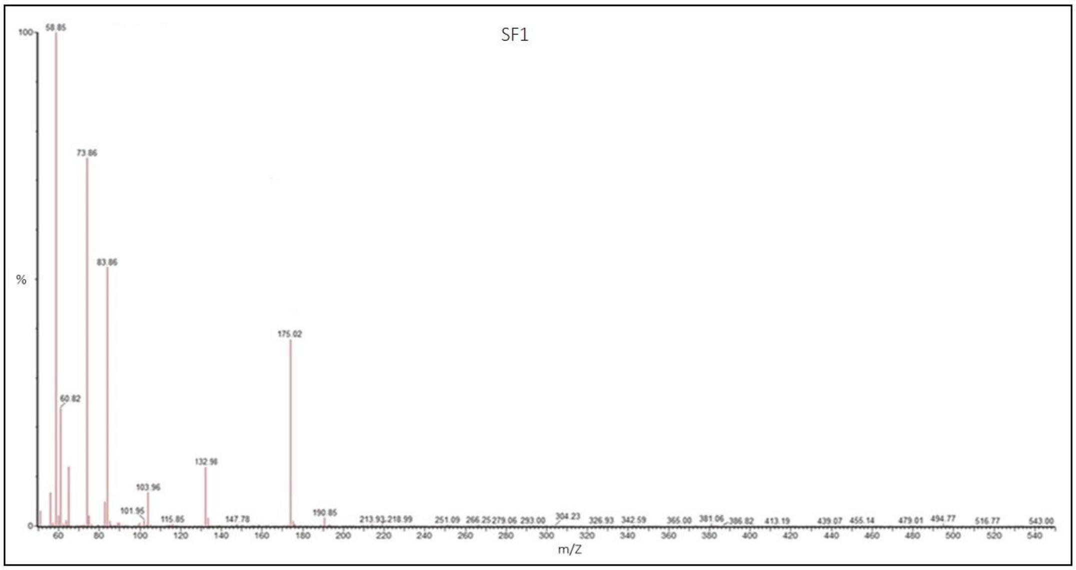
SF1
100
%
0
58.85
73.86
83.86
175.02
60.82
132.98
103.96
101.95
115.85
147.78
190.85
m/Z

B)

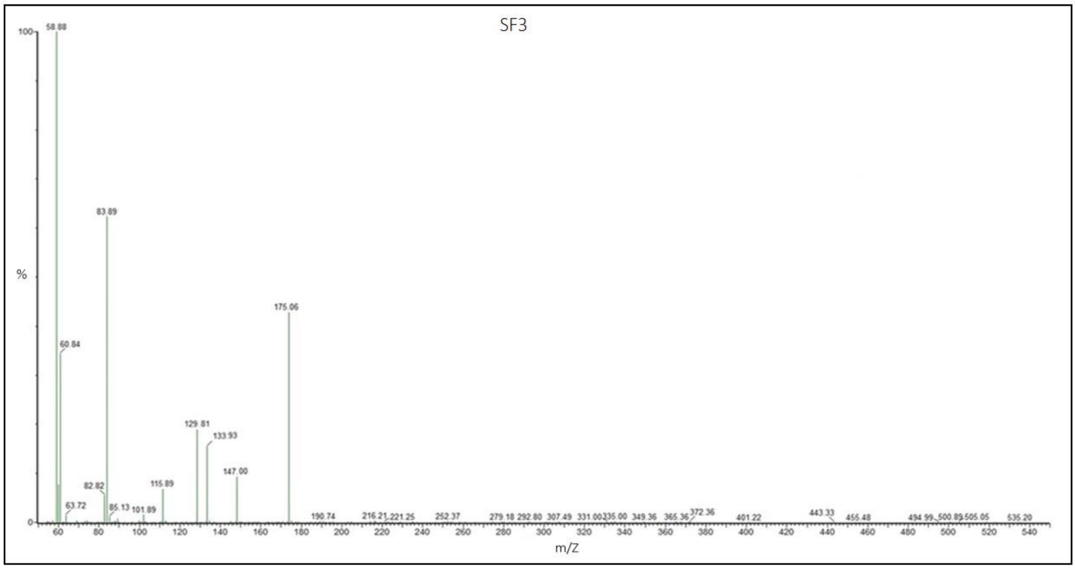
SF3
100
%
0
58.88
83.89
175.06
60.84
129.81
133.93
147.00
115.89
82.82
63.72
85.13
101.89
m/Z

C)

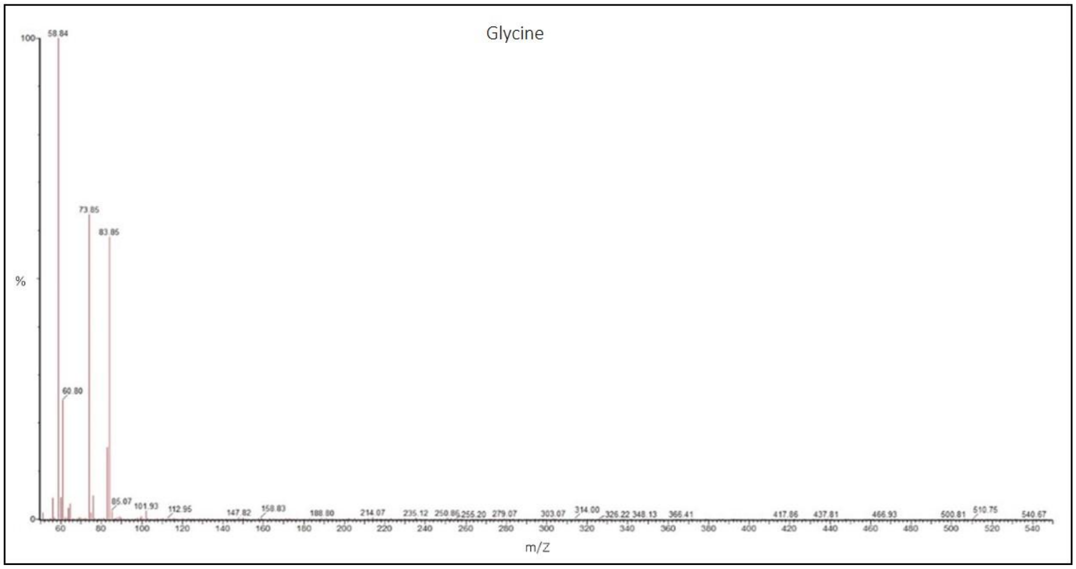
Glycine
100
%
0
58.84
73.85
83.85
60.80
85.07
101.93
112.95
m/Z

D)

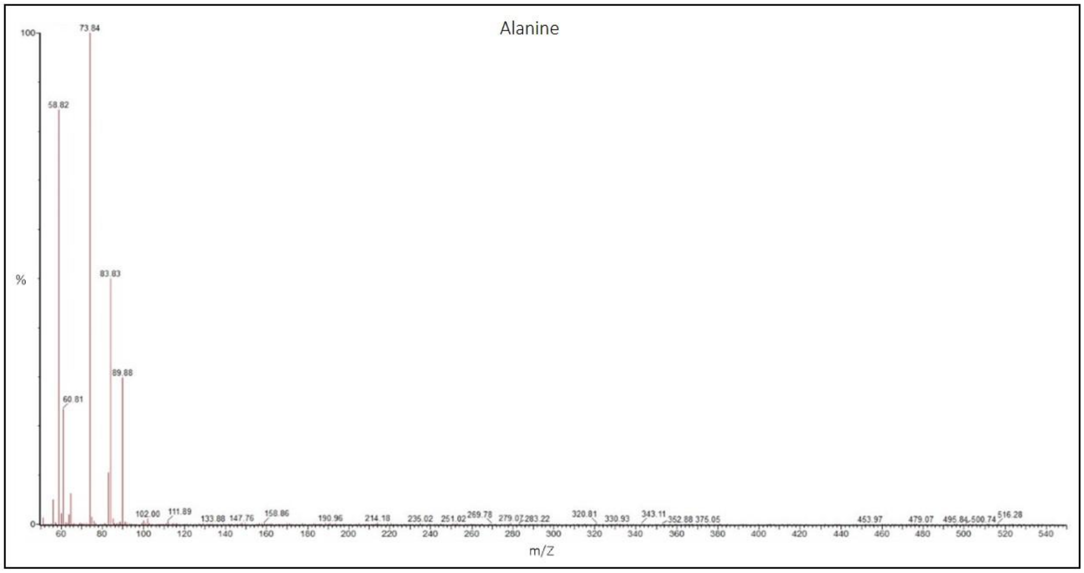
Alanine
100
%
0
73.84
58.82
83.83
89.88
60.81
m/Z

E)

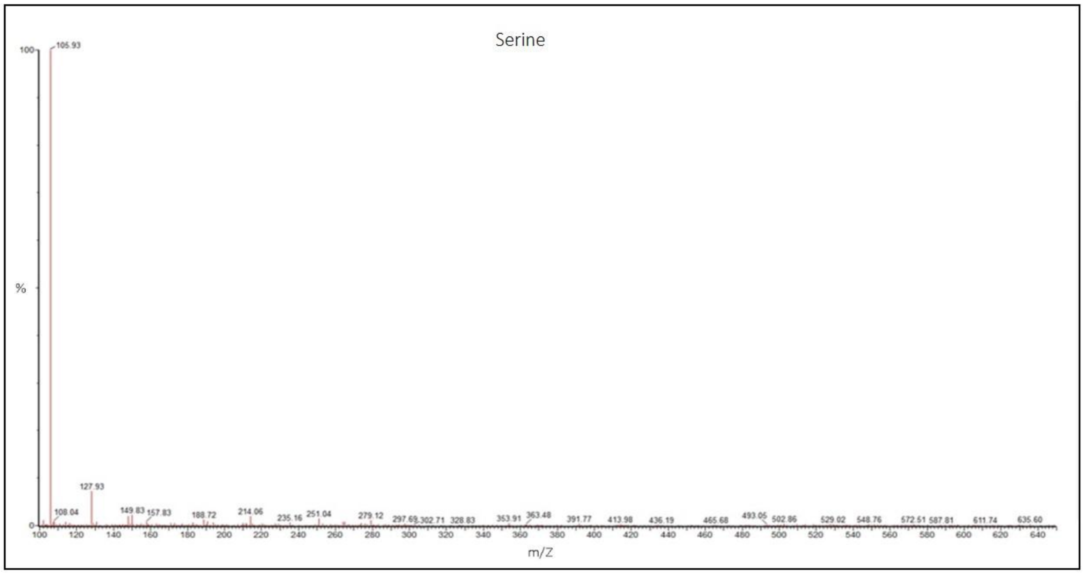
Serine
100
%
0
105.93
127.93
m/Z

F)

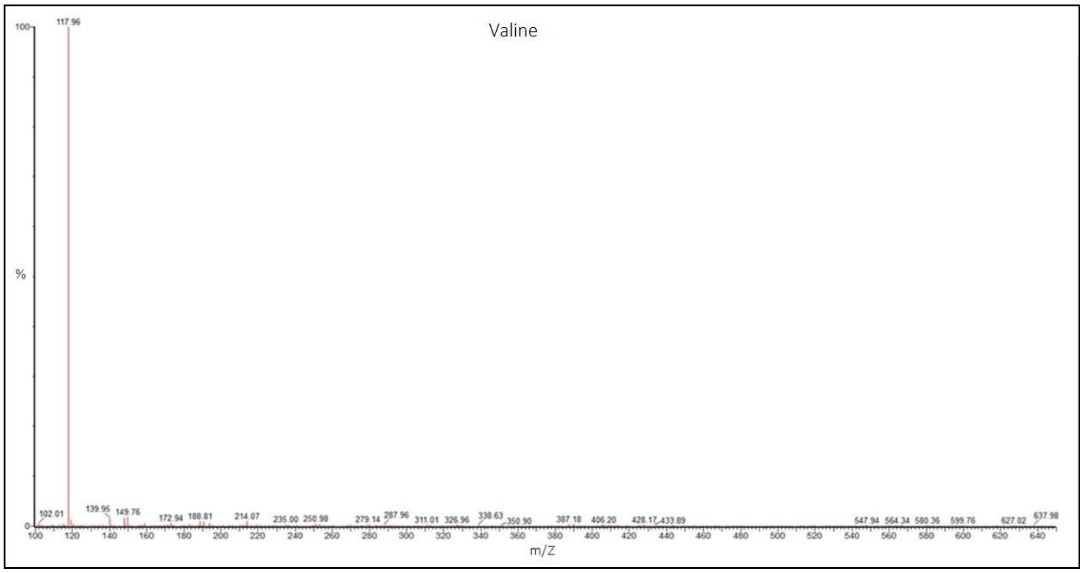
Valine
100
%
0
117.96
m/Z

G)

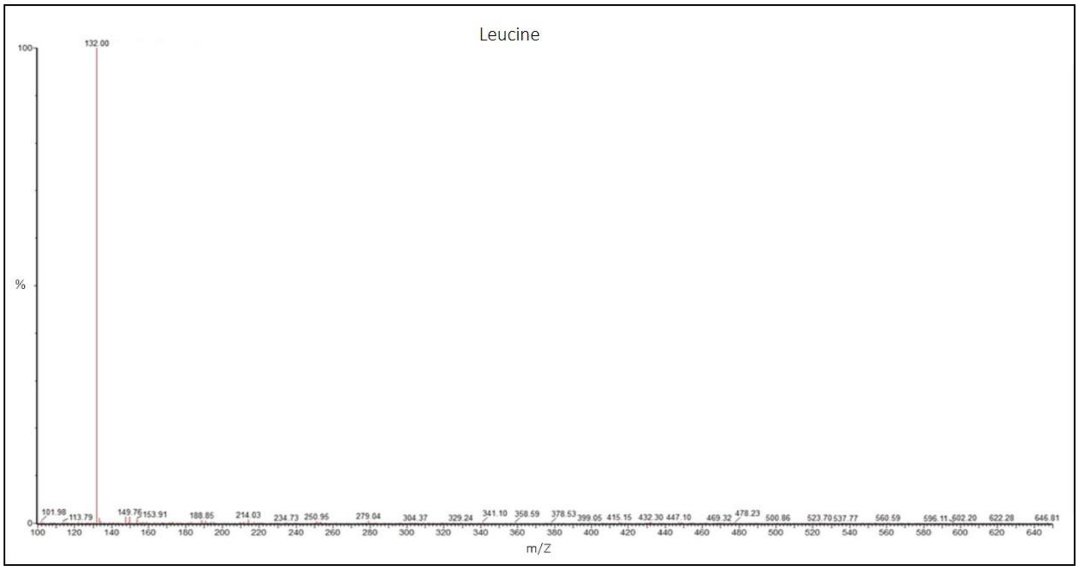

Leucine
100
%
0
132.00
101.98
113.79
149.76
153.91
188.85
214.03
234.73
250.95
279.04
304.37
329.24
341.10
368.59
378.53
399.05
415.15
432.30
447.10
469.32
478.23
500.86
523.70
537.77
560.59
596.11
602.20
622.28
646.81
100 120 140 160 180 200 220 240 260 280 300 320 340 360 380 400 420 440 460 480 500 520 540 560 580 600 620 640
m/Z

H)

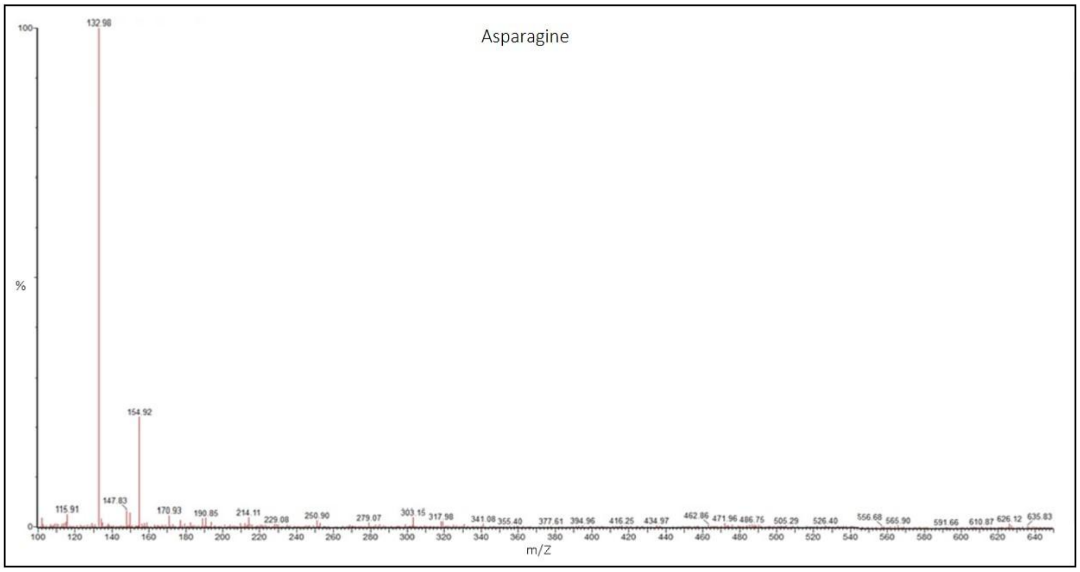

Asparagine
100
%
0
132.98
154.92
115.91
147.83
170.93
190.85
214.11
229.08
250.90
279.07
303.15
317.98
341.08
355.40
377.61
394.96
416.25
434.97
462.86
471.96
486.75
505.29
526.40
556.68
565.90
591.66
610.87
626.12
635.83
100 120 140 160 180 200 220 240 260 280 300 320 340 360 380 400 420 440 460 480 500 520 540 560 580 600 620 640
m/Z

I)

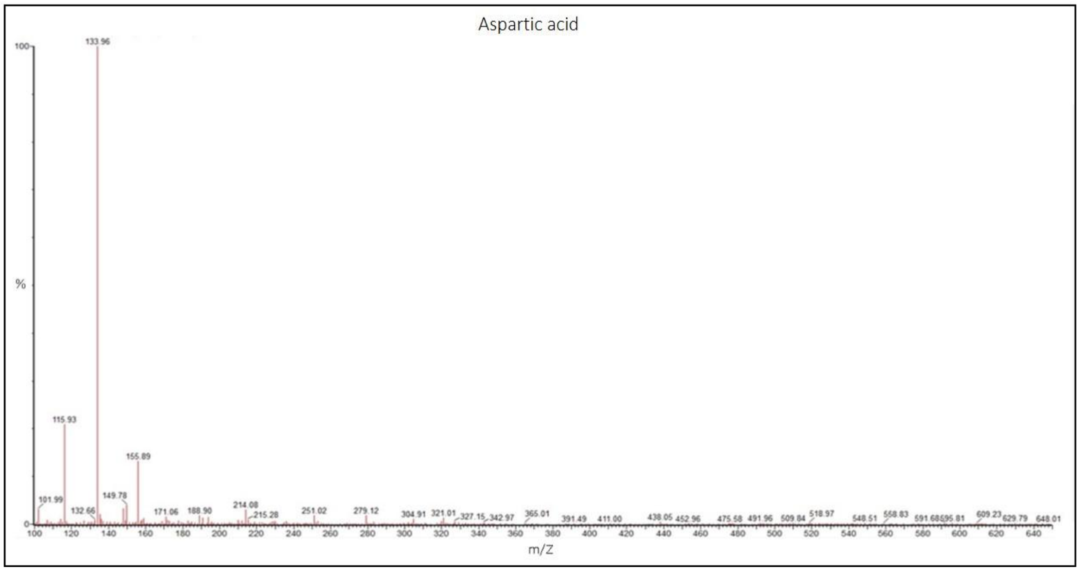

Aspartic acid
100
%
0
133.96
115.93
155.89
101.99
132.66
149.78
171.06
188.90
214.08
215.28
251.02
279.12
304.91
321.01
327.15
342.97
365.01
391.49
411.00
438.05
452.96
475.58
491.96
509.84
518.97
548.51
558.83
591.68
595.81
609.23
629.79
648.01
100 120 140 160 180 200 220 240 260 280 300 320 340 360 380 400 420 440 460 480 500 520 540 560 580 600 620 640
m/Z

J)

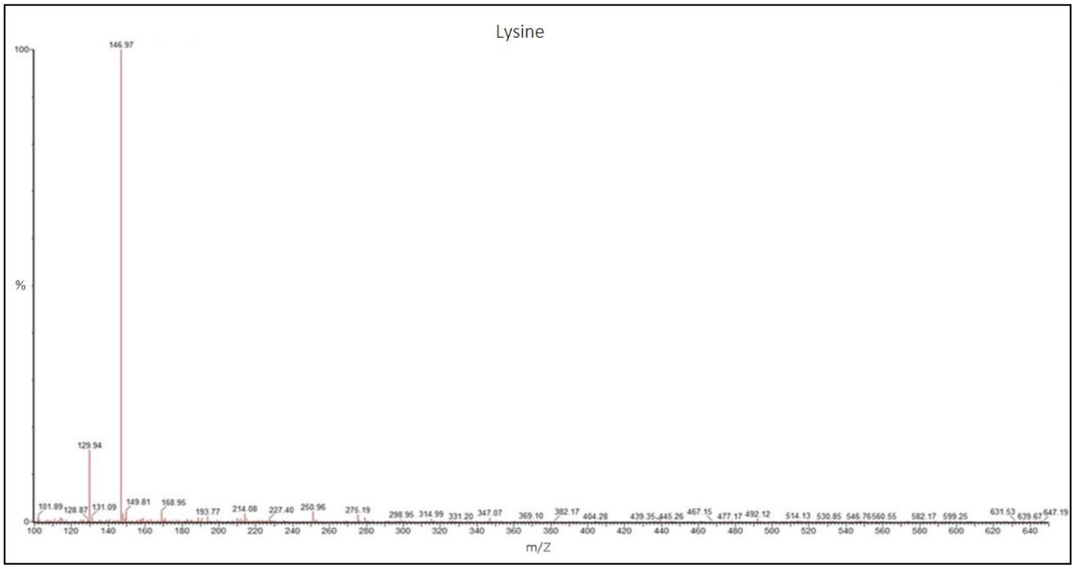
Lysine
100
146.97
%
129.94
101.89 128.87 131.09 149.81 168.95 193.77 214.08 227.40 250.96 275.19 298.95 314.99 331.20 347.07 369.10 382.17 404.28 439.35 445.26 467.15 477.17 492.12 514.13 530.85 546.76 560.55 582.17 599.25 631.53 639.67 647.19
0
100 120 140 160 180 200 220 240 260 280 300 320 340 360 380 400 420 440 460 480 500 520 540 560 580 600 620 640
m/Z

K)

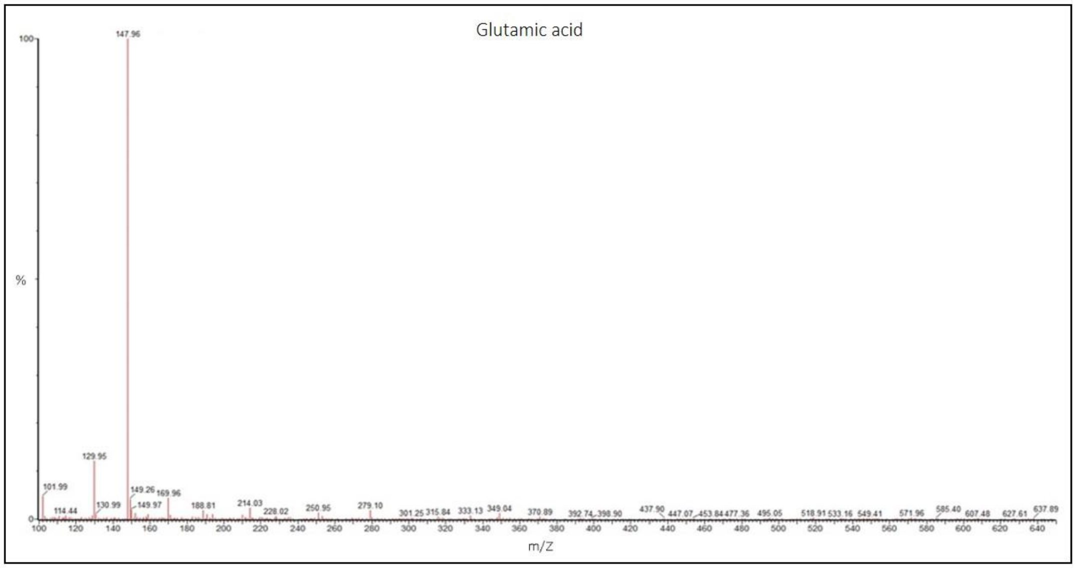
Glutamic acid
100
147.96
%
129.95
101.99 114.44 130.99 149.26 149.97 169.96 188.81 214.03 228.02 250.95 279.10 301.25 315.84 333.13 349.04 370.89 392.74 398.90 437.90 447.07 453.84 477.36 495.05 518.91 533.16 549.41 571.96 585.40 607.48 627.61 637.89
0
100 120 140 160 180 200 220 240 260 280 300 320 340 360 380 400 420 440 460 480 500 520 540 560 580 600 620 640
m/Z

L)

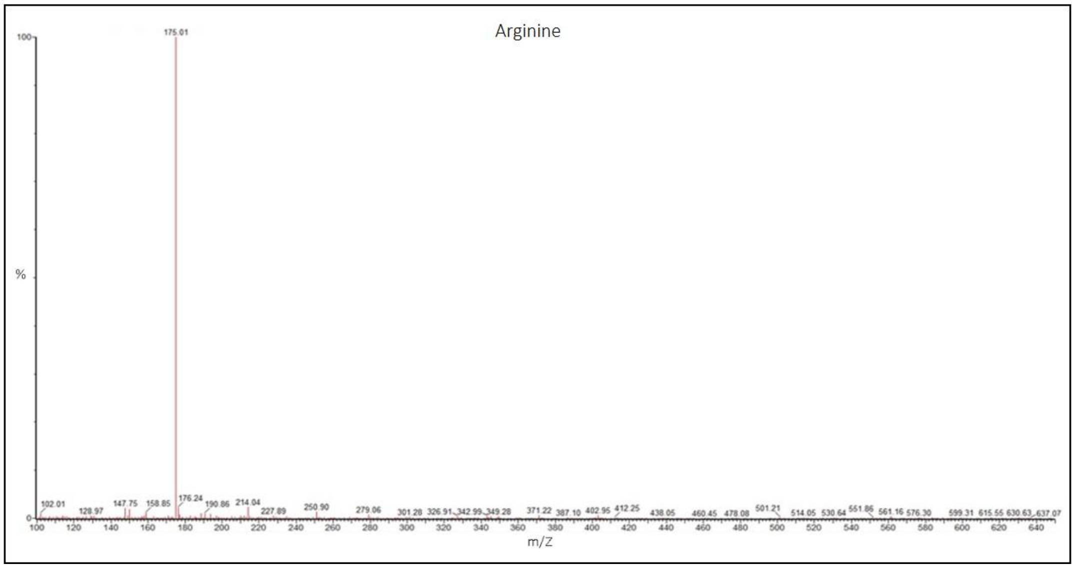
Arginine
100
175.01
%
102.01 128.97 147.75 158.85 176.24 190.86 214.04 227.89 250.90 279.06 301.28 326.91 342.99 349.28 371.22 387.10 402.95 412.25 438.05 460.45 478.08 501.21 514.05 530.64 551.86 561.16 576.30 599.31 615.55 630.63 637.07
0
100 120 140 160 180 200 220 240 260 280 300 320 340 360 380 400 420 440 460 480 500 520 540 560 580 600 620 640
m/Z

Supplementary Figure 3. Mass spectrometry. A) SF1 contains glycine, asparagine and arginine (peaks 58.85, 132.98 and 175.02, respectively). B) SF3 contains glycine, aspartic acid, lysine and arginine (peaks 58.88, 133.93, 147.00 and 175.06, respectively. Amino acid standards are shown in C to L: C) Glycine, D) Alanine, E) Serine, F) Valine, G) Leucine, H) Aspartic acid, I) Asparagine, J) Lysine, K) Glutamic acid and L) Arginine.
